# Dynamic Changes in Perioperative Cellular Inflammation and Acute Kidney Injury after Coronary Artery Bypass Grafting

**DOI:** 10.21470/1678-9741-2020-0163

**Published:** 2021

**Authors:** Hakan Parlar, Ali Ahmet Arıkan, Attila Önmez

**Affiliations:** 1 Department of Cardiovascular Surgery, Derince Research and Training Hospital, Kocaeli, Turkey.; 2 Department of Cardiovascular Surgery, Kocaeli University Medical Faculty, Kocaeli, Turkey.; 3 Department of Internal Medicine, Düzce University Medical Faculty, Düzce, Turkey.

**Keywords:** Coronary Artery Bypass, Acute Kidney Injury, Neutrophils, Lymphocytes, Platelet Count, Length of Stay, Patient Discharge.

## Abstract

**Introduction:**

This study investigated the role of the neutrophil-lymphocyte ratio (NLR), the perioperative changes in NLR (delta-NLR), the platelet-lymphocyte ratio (PLR), and the platelet count in predicting acute kidney injury (AKI) following coronary artery bypass grafting (CABG) during hospital stay.

**Methods:**

The records of 396 patients with preoperative creatinine < 1.5 mg/dl undergoing isolated CABG between October 2015 and October 2019 were reviewed retrospectively. Diagnosis of AKI was based on the Kidney Disease Improving Global Outcomes definition. Demographic data, operative data, in-hospital mortality, preoperative NLR, PLR, and platelet counts were compared between patients with (AKI group) and without (non-AKI group) postoperative AKI. Additionally, NLR, delta-NLR, and PLR values were calculated daily for the first four postoperative days. A “subsequent AKI group” was formed for the first four postoperative days by excluding patients diagnosed with AKI. The daily and overall predictivity of the markers for AKI are investigated.

**Results:**

AKI was present in 86 patients during the postoperative period, while 310 patients had normal postoperative renal functions. NLR, delta-NLR, and PLR on the first four postoperative days (*P*<0.001 for all) were significantly associated with the development of AKI in subsequent days. Multivariate analysis identified postoperative NLR (odds ratio 1.17, 95% confidence interval 1.11-1.23; *P*<0.001) as an independent predictor of AKI. PLR lost its significant association with AKI at the values measured at discharge from hospital (*P*>0.05).

**Conclusion:**

NLR values measured on the first four days postoperatively are a useful tool in predicting AKI during hospital stay following CABG.

**Table:** 

Abbreviations, acronyms & symbols				
**AKI**	**= Acute kidney injury**		**KDIGO**	**= Kidney Disease Improving Global Outcomes**
**AUC**	**= Area under the curve**		**LDL**	**= Low-density lipoprotein**
**BMI**	**= Body mass index**		**LVEF**	**= Left ventricular ejection fraction**
**BUN**	**= Blood urea nitrogen**		**MI**	**= Myocardial infarction**
**CABG**	**= Coronary artery bypass grafting**		**NLR**	**= Neutrophil-lymphocyte ratio**
**CI**	**= Confidence interval**		**OR**	**= Odds ratio**
**CPB**	**= Cardiopulmonary bypass**		**PLR**	**= Platelet-lymphocyte ratio**
**CRP**	**= C-reactive protein**		**Preop.**	**= Preoperative**
**CVA**	**= Cerebrovascular accident**		**ROC**	**= Receiver operating curve**
**EF**	**= Ejection fraction**		**SD**	**= Standard deviation**
**Hgb**	**= Hemoglobin**		**Sens.**	**= Sensitivity**
**IL**	**= Interleukin**		**Spec.**	**= Specificity**

## INTRODUCTION

Acute kidney injury (AKI) following cardiac surgery is a common complication which increases costs, morbidity, and mortality, even in cases in which renal failure does not occur^1,2^. The reported incidence of AKI following cardiac surgery is 5-30%, and cardiac surgery is the second most common cause of AKI in intensive care units^3,4^. The exact pathogenesis of post-cardiac surgery AKI is unclear. However, multiple factors, including systemic inflammation that may occur in the preoperative, intraoperative, and postoperative periods, will result in postoperative renal injury^5^. The early diagnosis of AKI can serve as a guide to treatment and improve prognosis. Neutrophil-lymphocyte ratio (NLR) and platelet-lymphocyte ratio (PLR) measured before surgery have been identified as simple markers for predicting AKI following cardiac surgery^2,6,7^. Urea or creatinine values measured in the first 48-72 hours after cardiac surgery are insufficient for demonstrating kidney damage^4,8^. Therefore, a reliable predictor of AKI is needed in the first few postoperative days.

An increase in neutrophil numbers and a decrease in lymphocyte and platelet counts occur immediately following cardiac surgery, and it may take months for all blood parameters to fully recover^9^. These dynamic changes are attributed to systemic inflammation, which is also a risk factor for AKI. The changes in NLR in the early postoperative period from preoperative values (delta-NLR) are reported to be a prognostic factor in predicting outcomes of surgery in cases of malignancy due to their association with systemic inflammation^10-12^. Earlier studies revealed an association between baseline and postoperative hematological indices and postoperative adverse outcomes, rather than the effect of acute changes in these parameters. The relationship between AKI and the delta-NLR in patients undergoing isolated coronary artery bypass grafting (CABG) has not been studied to date.

The fall of platelet count was related with development of AKI following CABG^13^. Paradoxically, higher PLR values on postoperative day one were also found to be related with AKI^2^. This caused suspicion about the predictive power of PLR in the postoperative days of CABG.

The aim of this study was to investigate the relationships between NLR, PLR, delta-NLR, platelet counts, and AKI during hospital stay of patients undergoing isolated CABG.

## METHODS

### Study Population

Approval for this study was granted by the local ethical committee, and all procedures were conducted in accordance with the principles set out in the Declaration of Helsinki. The medical records of 631 patients undergoing CABG between October 2015 and October 2019 were reviewed retrospectively.

Patients with low hemoglobin (Hgb) levels (≤ 10 g/dL), renal impairment (serum creatinine levels > 1.5 mg/dl or patients on dialysis), with active infection or active or chronic autoimmune disease, taking steroids or chemotherapeutic drugs, with moderate or severe valvular heart disease, scheduled for concomitant surgery, in a critical preoperative condition (requiring inotropic drug support or intra-aortic balloon pumping), with a left ventricular ejection fraction (LVEF) ≤ 30%, requiring respiratory support, individuals who were reoperated because of hemodynamic instability or bleeding, patients who were operated on a beating heart, and patients undergoing repeat CABG were excluded. Three hundred ninety-six patients aged between 38 and 79 years and who underwent isolated CABG with cardiopulmonary bypass (CPB) were finally enrolled. Patients’ demographic and clinical data were retrieved from the electronic hospital records and archives.

### Definitions

Characteristics such as age, gender, smoking history, diabetes, hypertension, hyperlipidemia, LVEF, pre- and postoperative laboratory parameters (Hgb levels, and leukocyte, neutrophil, lymphocyte, and platelet counts), serum creatinine, blood urea nitrogen (BUN), albumin, operative findings including duration of CPB and aortic cross-clamping, number of revascularized vessels, and in-hospital mortality were recorded. Hypertension was defined as blood pressure ≥ 140/90 mmHg or antihypertensive drug use, while smoking status was defined as positive if the individual concerned had not quit for at least the previous year. Diabetes was defined as a fasting blood glucose value ≥ 126 mg/dl or the use of antidiabetic medications, while hyperlipidemia was defined as a total cholesterol level > 220 mg/dl and low-density lipoprotein-cholesterol > 130 mg/dl or receipt of antihyperlipidemic drugs. Distal anastomoses were grouped as 1-3 and > 3 in number.

Diagnosis of AKI was based on the Kidney Disease Improving Global Outcomes (KDIGO) definition (serum creatinine rising by ≥ 0.3 mg/dl within 48 hours of surgery or rising to ≥ 1.5 times baseline values, or renal replacement therapy being required). Baseline serum creatinine was defined as the final concentration measured in the last week before surgery, while postoperative serum creatinine levels were measured daily following surgery^8^. Urine output was not employed since this can be affected by diuretics and/or intravenous fluids administered during and after surgery^2,8^. Patients who developed AKI following surgery during hospital stay were classified as the AKI group, and the day when AKI developed was noted. Those with normal postoperative renal functions were classified as the non-AKI group.

NLR and PLR values were first calculated. In order to show dynamic changes in NLR, delta-NLR was calculated by subtracting the preoperative NLR from the postoperative NLR (postoperative NLR-preoperative NLR)^10^. In order to analyze the daily predictivity of the parameters for the development of AKI in the first four postoperative days, patients who were diagnosed with AKI on or before the studied day were excluded from the AKI group and the “subsequent AKI group” was formed for postoperative days one, two, three, and four. The leukocyte, neutrophil, lymphocyte, platelet counts, NLR, delta-NLR, and PLR values were compared for every day between the “subsequent AKI group” and the “non-AKI group”. Additionally, to investigate the predictive value of the postoperative parameters for the occurrence of AKI, the method used by Kertai et al.^13^ was applied by calculating the nadir platelet and peak NLR, delta-NLR, and PLR values from the results obtained on the first four postoperative days. The minimum (nadir) platelet count was defined as the lowest in-hospital value measured in the first four postoperative days. Similarly, the maximum NLR, delta-NLR, and PLR values in the first four postoperative days were defined as peak NLR, delta-NLR or PLR. Patients preoperative demographic characteristics, laboratory parameters, and preoperative NLR, PLR, peak NLR, peak PLR, and nadir platelet values are compared between AKI and non-AKI groups.

The leukocyte, neutrophil, lymphocyte, platelet counts, NLR, delta-NLR, and PLR values were calculated for the first, second, third, and fourth postoperative days (NLR 1, 2, 3, and 4, delta-NLR 1, 2, 3, and 4, PLR 1, 2, 3, and 4) and on the day of discharge from hospital. Approximately 5 to 7 ml of venous blood samples were added to a sterile tube with ethylenediaminetetraacetic acid. An automated blood count device (Abbott CELL-DYN 3700; Abbott Laboratory, Abbott Park, Illinois, United States of America) was employed to calculate hematological parameters following a one-hour waiting period.

### Operative Technique

All procedures were performed by the same surgical team. Following induction of general anesthesia, median sternotomy was performed, and left internal thoracic artery and saphenous venous grafts were prepared. Following systemic heparin administration (300 IU/kg), aortic and venous cannulations were performed and CPB was instituted. The activated clotting time was maintained over 450 seconds during the procedures, moderate hypothermia (28-32 ºC) is used. CPB flow was maintained at 2.2-2.5 l/min/m^2^, and mean blood pressure was held at between 50 and 80 mmHg. Hematocrit levels of 20-25% were maintained during CPB. Cross-clamping was applied to the ascending aorta, followed by antegrade hypothermic and hyperkalemic blood cardioplegia. Distal anastomoses were performed during aortic cross-clamping, while proximal anastomoses were performed on beating heart over the ascending aorta with a lateral clamp.

After surgery, all patients were moved to the intensive care unit. Patients were extubated following the restoration of normal orientation and cooperation if their hemodynamic and respiratory functions were recovered. If no contraindication was determined, all patients were started on 50 mg/day oral metoprolol after the first postoperative day.

### Statistics

Statistical analysis was performed on IBM Corp. Released 2013, IBM SPSS Statistics for Windows, Version 22.0, Armonk, NY: IBM Corp. Data exhibiting normal distribution were expressed as mean ± standard deviation, and those without normal distribution as median values (minimum-maximum). Data obtained by counting were expressed as percentages (%). Normality of distribution was assessed using a histogram or the Kolmogorov-Smirnov test, while homogeneity of distribution was assessed using Levene’s test for equality of variance. Intergroup comparisons were performed using Student’s t-test in the event of normal and homogenous distribution, or with the Mann-Whitney U test if distribution was not normal and homogeneous. Differences between the groups were evaluated by parametric or non-parametric Pearson’s Chi-Square test or Fisher’s exact test, depending on whether or not the distribution was parametric. The effects of the preoperative risk factors thought to affect the development of AKI (including peak NLR, delta-NLR, PLR, and nadir platelet counts) were investigated using univariate logistic regression analysis. The multiple effects of the risk factors identified as being effective or possibly effective in the prediction of AKI at univariate statistical analysis were then investigated using multivariate logistic regression analysis. The development of AKI on subsequent days was investigated using univariate regression analysis in terms of NLR, delta-NLR, and PLR on the first, second, third, and fourth postoperative days. Odds ratio, 95% confidence interval, and level of significance were determined for each risk factor. *P*-values < 0.05 were regarded as statistically significant. Receiver operating curve (ROC) analysis was used to compute the sensitivity and the specificity of preoperative NLR, PLR, peak NLR, peak delta-NLR, and peak PLR in predicting in-hospital AKI. The sensitivity and specificity of NLR, delta-NLR, and PLR in the first, second, third, and fourth postoperative days to predict AKI on the subsequent days were computed using ROC analysis.

Results were considered statistically significant at *P*<0.05.

## RESULTS

Three hundred ninety-six patients (mean age 62.2±9 years, 78.7% men) were included into the study. Mean hospitalization time was 7.27±1.73 days. AKI, diagnosed based on the KDIGO classification, was present in 86 patients (Group 1, AKI 22%), while 310 patients had normal postoperative renal functions (Group 2, non-AKI, 78%). Seventeen patients were diagnosed with AKI on postoperative day one. Sixty-nine patients developed AKI after postoperative day one, 21 after day two, nine after day three, and four after day four, and these patients’ values were used to analyze development of subsequent AKI for any specific postoperative day. None of these patients required renal replacement therapy. Demographic characteristics and preoperative results and operative data are summarized in Table 1. Age (*P*=0.014), preoperative creatinine (*P*<0.001), BUN (*P*=0.002), albumin (*P*=0.016), lymphocyte count (*P*<0.001), preoperative NLR (*P*<0.001), and preoperative PLR (*P*<0.001) levels differed significantly between the groups.

**Table 1:** Demographics, preoperative laboratory results, NLR and PLR values, and operative data.

Demographics	Non-AKI (n=310)	AKI (n=86)	*P*-value
Age (years) (mean±SD)	60.6±8.8	63±8.6	0.014
Sex, male, n (%)	243 (78)	69 (80)	0.767
Weight, kg	78.9±16	79.1±14	0.881
BMI, kg/m^2^	27,7±4.4	28.1±4.6	0.475
Diabetes mellitus, n (%)	95 (30.6)	31 (34.8)	0.512
Hypertension, n (%)	199 (64.1)	58 (67.4)	0.724
Smoking, n (%)	122 (39.3)	38 (44.1)	0.641
Hyperlipidemia, n (%)	138 (42.3)	33 (37.5)	0.475
Preoperative EF, %	58.3±9	56±10	0.153
Postoperative EF, %	57±8	57±10	0.910
History of atrial fibrillation, n (%)	129 (41.6)	29 (33.7)	0.591
History of CVA, n (%)	11 (356)	5 (5,8)	0.432
History of MI, n (%)	69 (22,2)	21 (24,4)	0.332
**Preoperative measurements**			
Creatinine, mg/dl	0,88±0,18	0,99±0,28	< 0.001
BUN	16.8±6.1	20.8±7.7	0.002
Albumin	3.8±0.4	3.7±0.4	0.016
Total cholesterol	128 (80-404)	126 (70-319)	0.936
LDL	128±36,8	118±42,6	0.359
Triglyceride	123 (40-675)	128 (31-441)	0.636
CRP	8.1 (0.2-145)	9.5 (1.7-95)	0.446
Hemoglobin (mg/dl)	13.7±1	13.6±1.2	0.311
Leukocyte count (´ 10^3^/µL)	8.55±2.58	8.06±2.36	0.078
Neutrophil (´ 10^3^/µL)	5.36±1.85	5.41±1.88	0.81
Lymphocyte (´ 10^3^/µL)	2.32±0.79	1.84±0.77	< 0.001
Platelet (´ 10^3^/µL)	224.92±62.42	238.40±72.58	0.088
NLR	2.51±0.85	3.32±1.15	< 0.001
PLR	107.51±31.25	154.99±43.6	< 0.001
**Operative data**			
Cross-clamping time (min)	62±19	60±21	0.301
CPB time (min)	109±29	107±33	0.729
CABG 1-3 (%)	138 (44.5)	42 (48.8)	
CABG > 3 (%)	172 (55.5)	44 (51.2)	
In-hospital mortality, n (%)	5 (1.61)	1 (1.47)	1.000

Data are expressed as mean±SD and median (minimum-maximum); significant results are shown in bold typeface. AKI=acute kidney injury; BMI=body mass index; BUN=blood urea nitrogen; CABG=coronary artery bypass grafting; CPB=cardiopulmonary bypass; CRP=C-reactive protein; CVA=cerebrovascular accident; EF=ejection fraction; LDL=low-density lipoprotein; MI=myocardial infarction; NLR=neutrophil-lymphocyte ratio; PLR=platelet-lymphocyte ratio; SD=standard deviation

Comparisons of the subsequent AKI group with the non-AKI group revealed significantly higher leukocyte counts on postoperative days two, three, and four (*P*<0.001 all), significantly higher neutrophil counts on postoperative days one, two, three, and four (*P*=0.008, *P*<0.001, *P*<0.001, and *P*<0.001, respectively), significantly lower lymphocyte counts on postoperative days one, two, and three (*P*<0.001, *P*<0.001, and *P*=0.001, respectively), and significantly lower platelet counts on postoperative days one, two, three, and four (*P*<0.001, *P*<0.001, *P*<0.001, and *P*=0.006, respectively). NLR, delta-NLR, and PLR values on postoperative days one, two, three, and four were significantly higher (*P*<0.001, *P*<0.001, *P*<0.001, and *P*=0.001, respectively; all) in the subsequent AKI group. This indicates that the profile of cellular inflammation changes in the first four postoperative days, and that an increase in NLR, delta-NLR, and PLR values occurs at least one day prior to diagnosis of AKI (Table 2).

Peak NLR and peak PLR values on the first four postoperative days were significantly higher (*P*<0.001 for both) among patients who developed AKI compared to the non-AKI group. Nadir platelet counts were significantly lower (*P*=0.001) among patients who developed AKI (Table 2).

**Table 2:** Hematological parameters for the prediction of AKI for each postoperative day.

Postoperative measurements	Day*	Non-AKI	Subsequent AKI	*P*-value
Hemoglobin (mg/dl)	1	9.3 (7.4-12.1)	8.9 (7.6-11.9)	0.410
Leukocyte count (´ 10^3^/µL)	1	12.68±3.33	13.13±4.35	0.338
	2	11.04±3.28	14.14±3.15	< 0.001
	3	11.01±3.17	15.76±1.7	< 0.001
	4	10.45±3.2	16.05±1.57	< 0.001
Neutrophil count (´ 10^3^/µL)	1	11.04±3.28	12.28±3.03	< 0.001
	2	10.50±2.93	13.59±1.58	0.008
	3	8.48±2.83	14.44±2.70	< 0.001
	4	8.23±2.93	15.45±2.43	< 0.001
Lymphocyte count (´ 10^3^/µL)	1	0.93±0.39	0.58±0.22	< 0.001
	2	0,83 (0.03-2.63)	0.28 (0.25-0.85)	< 0.001
	3	1.40 (0.01-3.6)	0.45 (0,30-2.1)	0.001
	4	1.51±0.06	0.75±0.09	0.139
Platelet count (´ 10^3^/µL)	1	179.43±49.41	156.07±48.29	< 0.001
	2	176.69±51.25	131.20±30.48	< 0.001
	3	174.04±52.18	148.11±47.27	< 0.001
	4	283.32±105.36	134.74±50.72	0.006
NLR	1	12.91±6.24	23.38±8.48	< 0.001
	2	12.95±6.24	32.98±11.95	< 0.001
	3	5.65 (1.97-119)	52.66 (4.91-58.76)	< 0.001
	4	5.3 (-4-78)	61.8 (51.7-72.6)	0.001
Delta-NLR	1	10.28±6.21	20.09±8.55	< 0.001
	2	9.24 (-3.38-41,72)	39.5 (6.18-45.4)	< 0.001
	3	3.22 (-2-115.69)	48.16 (2.96-51.26)	< 0.001
	4	2.89 (-7.58-74.69)	57.3 (37.5-61.4)	0.001
PLR	1	217.84±91.4	288.39±89.6	< 0.001
	2	215.26±97.56	362.31±114.16	< 0.001
	3	117.17 (22.5-2490)	446.66 (86.19-6357.56)	< 0.001
	4	185.89 (-112-2982.66)	467.6 (366.4-845.6)	0.001
	**Days** **	**Non-AKI**	**AKI**	***P*-value**
Peak NLR	01/4	12.20 (2.77-119)	22.31 (17-61.8)	< 0.001
Peak delta-NLR	01/4	11.9 (0-115)	20.82 (15.7-58.9)	< 0.001
Peak PLR	01/4	250.71 (87.50-2982.66)	332.05 (107.57-1601.6)	< 0.001
Nadir platelet count	01/4	160.25 (65-320)	141 (31-264)	0.001

* The postoperative day when the hematological parameters were obtained. The number of patients who are in the subsequent AKI group were: n=69 for day one, n=21 for day two, n=9 for day three, and n=4 for day four, while no change occurred in the non-AKI group (n=320).

** Nadir platelet counts and peak NLR, delta-NLR, and PLR values in the first four days after surgery are compared with the occurrence of AKI during hospital stay. AKI=acute kidney injury; NLR=neutrophil-lymphocyte ratio; PLR=platelet-lymphocyte ratio

Leukocyte, neutrophil, lymphocyte, and platelet counts, and NLR, delta-NLR, and PLR values on the day of discharge were compared among patients with and without occurrence of AKI during their hospital stays. The relations between leukocyte count and PLR values and the presence of AKI had lost their significance by the day of discharge from hospital (Table 3).

**Table 3:** The relation between the parameters and AKI at the day of discharge.

Parameters	Non-AKI (n=310)	AKI (n=86)	*P*-value
Leukocyte (´ 10^3^/µl)	10.25±2.88	10.77±2.66	0.138
Neutrophil (´ 10^3^/µl)	6.87±2.4	7.77±2.64	0.002
Lymphocyte (´ 10^3^/µl)	2.12±0.74	1.8±0.83	0.001
Platelet (´ 10^3^/µl)	295.32±97.01	246±98.4	< 0.001
NLR	3.15 (0.44-17.33)	4 (0.44-13.75)	< 0.001
PLR	152.4±66.09	145.77±58.1	0.402
Delta-NLR	0.88 (-4.63-14.58)	1.16 (-7.39-9.25)	0.045

AKI=acute kidney injury; NLR=neutrophil-lymphocyte ratio; PLR=platelet-lymphocyte ratio

Multivariate analysis of those variables found to be statistically significant at univariate analysis identified preoperative creatinine (*P*=0.001), preoperative albumin (*P*=0.03), preoperative leukocyte (*P*=0.018), preoperative NLR (*P*=0.007), and postoperative peak NLR (*P*<0.001) as independent predictors of postoperative AKI (Table 4).

**Table 4:** Univariate and multivariate regression analyses of risk factors for postoperative AKI.

Variables	Unadjusted OR (95% CI)	*P*-value	Adjusted OR (95% CI)	*P*-value
Age	1.04 (1.01-1.06)	0.014	-	-
Gender	0.89 (0.49-1.62)	0.711	-	-
Hypertension	1.234 (0.76-2.02)	0.391	-	-
Diabetes mellitus	0.82 (0.49-1.36)	0.455	-	-
Preoperative EF	0.98 (0.95-1.01)	0,154	-	-
Preoperative creatinine	15.72 (4.53-54.48)	< 0.001	12.58 (2.81-55.83)	0.001
Albumin	0.46 (0.24-0.87)	0.017	0.41 (0.18-0.91)	0.03
CRP	0.99 (0.98-1.01)	0.853		
Cross-clamping time	0.99 (0.98-1.00)	0.301	-	-
CPB time	0.99 (0.99-1.01)	0.728	-	-
Preoperative hemoglobin	0.87 (0.68-1.10)	0.87	-	-
Preoperative leukocyte	1.00 (1.00-1.01)	0.002	1.00 (1.00-1.01)	0.018
Preoperative NLR	1.49 (1.28-1.79)	< 0.001	1.37 (0.09-1.72)	0.007
Preoperative PLR	1.01 (1.00-1.02)	< 0.001		-
Postoperative peak NLR	1.14 (1.10-1.19)	< 0.001	1.17 (1.11-1.23)	< 0.001
Peak delta-NLR	1.12 (1.08-1.15)	< 0.001	-	-
Postoperative peak PLR	1.01 (1.00-1.01)	0.003	-	-
Postoperative nadir platelet	1.00 (0.99-1.00)	< 0.001	-	-

AKI=acute kidney injury; CI=confidence interval; CPB=cardiopulmonary bypass; CRP=C-reactive protein; EF=ejection fraction; NLR=neutrophil-lymphocyte ratio; OR=odds ratio; PLR=platelet-lymphocyte ratio

Univariate analysis results for NLR, delta-NLR, and PLR values on postoperative days one, two, three, and four in terms of prediction of subsequent AKI for each postoperative day are summarized in Table 5.

**Table 5:** Univariate regression analyses of the hematological indices for the prediction of AKI for each postoperative day.

Variables		Unadjusted OR (95% CI)	*P*-value
***For AKI after postoperative day 1***	NLR	1.19 (1.14-1.28)	< 0.001
	Delta-NLR	1.18 (1.13-1.24)	< 0.001
	PLR	1.01 (1.00-1.01)	< 0.001
***For AKI after postoperative day 2***	NLR	1.28 (1.14-1.29)	< 0.001
	Delta-NLR	1.21 (1.14-1.29)	< 0.001
	PLR	1.10 (1.00-1.02)	< 0.001
***For AKI after postoperative day 3***	NLR	1.15 (1.07-1.16)	< 0.001
	Delta-NLR	1.12 (1.07-1.16)	< 0.001
	PLR	1.00 (1.00-1.01)	0.037
***For AKI after postoperative day 4***	NLR	1.15 (1.05-1.24)	< 0.001
	Delta-NLR	1.15 (1.07-1.24)	< 0.001
	PLR	1.00 (1.00-1.00)	0.041

AKI=acute kidney injury; CI=confidence interval; NLR=neutrophil-lymphocyte ratio; OR=odds ratio; PLR=platelet-lymphocyte ratio

**Fig. 1:**
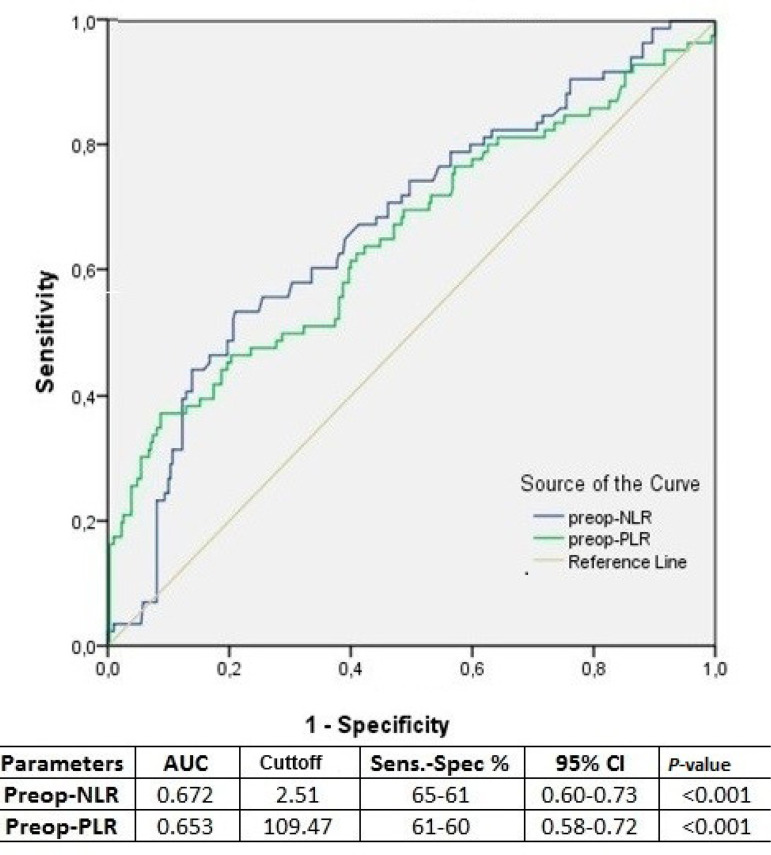
Receiver operating curve analysis for preoperative (preop.) neutrophil-lymphocyte ratio (NLR), delta-NLR, and plateletlymphocyte ratio (PLR) values regarding occurrence of postoperative acute kidney injury during hospital stay. AUC=area under the curve; CI=confidence interval; Sens.=sensitivity; Spec.=specificity

**Fig. 2:**
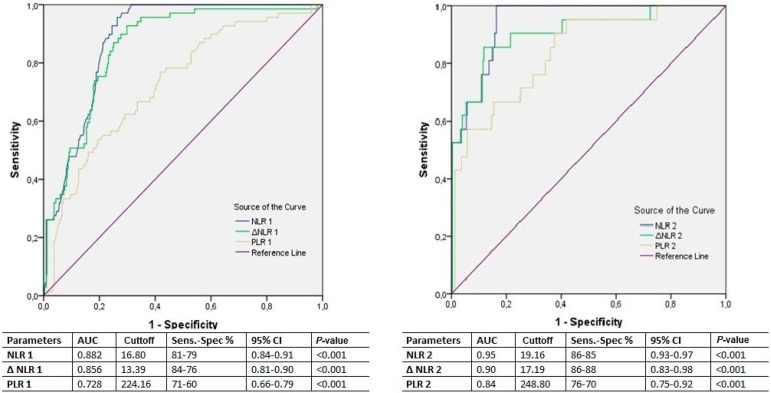
Receiver operating curve analysis for postoperative neutrophil-lymphocyte ratio (NLR), delta(Δ)-NLR, and platelet-lymphocyte ratio (PLR) values for postoperative days one and two, regarding occurrence of acute kidney injury on the subsequent days of hospital stay. AUC=area under the curve; CI=confidence interval; Sens.=sensitivity; Spec.=specificity

ROC curves were calculated to analyze the predictivity of preoperative NLR and PLR (Figure 1). Also, ROC curves were calculated for NLR, delta-NLR, and PLR on postoperative days one and two to determine their predictivity of AKI on subsequent days (Figure 2).

**Fig. 3:**
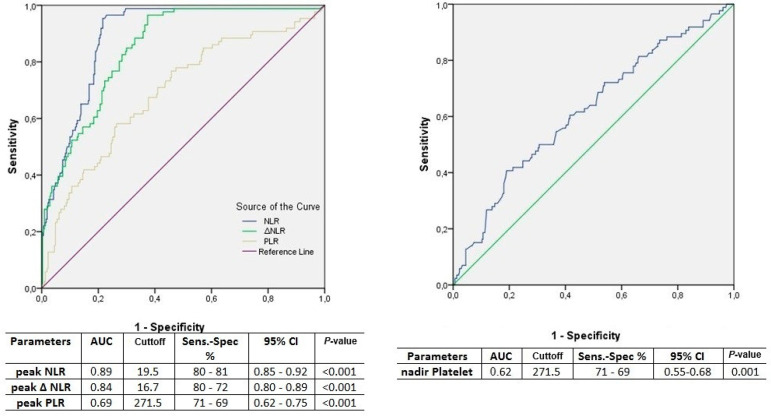
Receiver operating curve analysis of the peak neutrophil-lymphocyte ratio (NLR), peak delta(Δ)-NLR, and nadir platelet-lymphocyte ratio (PLR) values for the first four postoperative days regarding the occurrence of postoperative acute kidney injury during hospital stay. AUC=area under the curve; CI=confidence interval; Sens.=sensitivity; Spec.=specificity

ROC curve analysis results for NLR 3, delta-NLR 3, PLR 3, NLR 4, delta-NLR 4, and PLR 4 in terms of prediction of AKI on subsequent days are summarized in Table 6. The results of ROC curve analysis in predicting postoperative AKI for peak NLR, peak delta-NLR, and peak PLR are shown in Figure 3.

## DISCUSSION

This study shows the value of NLR, delta-NLR, and PLR measured on the first four days after CABG in terms of prediction of in-hospital AKI on subsequent days. Postoperative NLR values on the first four days following CABG emerged as the most reliable and independent predictors of in-hospital AKI.

The pathophysiology of AKI following cardiac surgery is multifactorial and includes inflammation, ischemia-reperfusion injury, operative trauma, blood exposed to the artificial surface of the CPB circuit, neurohormonal activation, metabolic factors, and oxidative stress^3,4,14^. Neutrophil activation plays a crucial role in the initiation and progression of inflammation and is implicated in reperfusion injury, and CPB itself is associated with neutrophil activation and an augmented neutrophil count^9,15^. Neutrophils play a limited role in atherogenesis, but lymphocytes are involved in the development and progression of atherosclerosis and are inversely associated with inflammation. A reduced lymphocyte count reflects physiological stress, and an increased cardiovascular risk and mortality^15,16^. CPB-induced inflammation increases average neutrophil values, which rapidly reach their peak on the second day after surgery, while lymphocytes decrease and achieve their lowest value on the first day after surgery^9^. Thrombocytosis is an important factor in the pathophysiology of atherosclerosis, and a reduction in platelet counts is observed following CPB^15,16^. NLR and PLR are easily available, inexpensive markers and are positively correlated with such inflammatory markers as tumor necrosis factor-α and interleukin (IL)-6^17^.

NLR and PLR values measured at the preoperative period have been reported to predict AKI following cardiac surgery^1,2^ The present study provides additional evidence that postoperative AKI is associated with elevated preoperative NLR and PLR values. One meta-analysis investigating NLR for the prediction of AKI estimated an area under the curve (AUC) of 0.65 in a cardiac surgery subgroup^7^. Similarly, a cutoff value of 2.65 (sensitivity 66.1%, specificity 64.7%) with an AUC of 0.69 has been reported for the predictivity of preoperative NLR^2^.

In the present study, ROC analysis yielded a similar result for preoperative NLR (AUC: 0.67) with a similar cutoff value of 2.51 (65% sensitivity, 61%specificity) (Figure 1). Additionally, preoperative NLR emerged as an independent predictor of postoperative AKI (Table 4).

Increased NLR can be related to the complexity of atherosclerosis or lower creatinine clearance levels, as previously reported. Preoperative NLR has been identified as a predictor for mortality and cardiovascular events following CABG^15,18^. However, the body is subject to an emerging postoperative inflammatory response as well as to the existing inflammatory burden of the preoperative period.

The postoperative NLR, delta-NLR, and PLR values were analyzed for their daily predictivity as well as the predictivity of their peak values. The later AKI developed, the higher were the NLR, delta-NLR, and PLR values prior to development of AKI (Table 2). These results suggest that a greater inflammatory response is triggered in patients who will develop AKI in later days after surgery. NLR and delta-NLR were significant for all four postoperative days at univariate logistic regression analysis (*P*<0.001 all). Meanwhile, the significance of P-values of PLR in predicting AKI diminished during the first four days (*P*<0.001, *P*<0.001, *P*=0.037, *P*=0.041, respectively) (Table 5). Daily postoperative NLR exhibited the highest predictivity for postoperative AKI at univariate analysis and ROC analysis (Table 5, Table 6, and Figure 2). In ROC analysis of daily NLR, delta-NLR, and PLR values for the occurrence of AKI, the AUC values augmented with the advance of days (Table 6). These values show that a high risk of AKI exists in patients with high cellular inflammation levels after the second day of surgery. However, the number of patients developing AKI after the third and fourth days were decreased (nine and four, respectively), this might have affected the very high AUC, sensitivity, and specificity of the measured parameters.

The peak NLR, delta-NLR, and PLR as well as nadir platelet counts of the first four postoperative days were compared in terms of the occurrence of AKI until discharge from hospital. Peak NLR emerged as an independent predictor of AKI (Table 4). ROC analysis revealed the highest AUC for NLR, a cutoff value of 19.5 exhibiting 80% sensitivity and 81% specificity. This threshold could be used to predict overall risk of AKI in the first four postoperative days.

Delta-NLR is used to demonstrate the dynamic change in NLR from its initial values to post-treatment values. This new marker is directly related to the effect of the treatment applied. The relationship between inflammation and delta-NLR has previously been studied in other surgical patient groups^10-12^. This study shows that delta-NLR is strongly related with AKI and delta-NLR values were significantly augmented prior the development of AKI. However, NLR values were superior to delta-NLR in predicting AKI as an independent predictor following CABG.

Surgery triggered an inflammatory response among all patients, which was measured with augmented postoperative NLR values comparing to preoperative baseline values (Table 1 vs. Table 2). By considering the NLR and delta-NLR values of patients who developed AKI during the postoperative days, the measured values were augmented for every day. The NLR and delta-NLR values obtained in the later days of the postoperative course as well as calculated cutoff values were higher than in the previous days. These results show that other factors than surgery alone might be involved in the development of AKI with the ongoing days. Additionally, in patients who did not developed AKI, the NLR and delta-NLR values decreased constantly with the course of time (Table 2, Table 6, and Figure 2). The difference in the parameters of cellular inflammation among patients with and without AKI cannot be attributed to the operation itself, since the CPB and cross-clamping times were similar among the groups. It was due to the augmented response of the body.

Platelet counts have previously been analyzed in order to evaluate their predictivity for the development of AKI following CABG, using nadir platelet counts^13^. Fall in platelet counts was also associated with AKI in the present study. However, platelet counts were not superior to NLR in terms of prediction of AKI based on AUC in the ROC (Table 4 and Figure 3).

While preoperative platelet counts were higher in patients with AKI, this was not statistically significant (Table 1). In contrast, as reported previously, platelet counts decreased progressively during the first four postoperative days and were significantly lower prior to the development of AKI compared to the non-AKI patients^9^. Additionally, platelet counts were significantly lower in patients with AKI on the day of discharge in accordance with previous reports^13^.

PLR values measured at the preoperative period and on the postoperative day one have been reported to predict AKI following cardiac surgery^2^. Concerns about the predictivity of PLR as an inflammatory marker following CABG exist due to an expected fall in platelet levels following surgery^19^. Additionally, an increased risk for AKI has been reported with a decrease in platelet counts^13^. However, due to a greater fall in lymphocyte levels, PLR was correlated with the presence or absence of AKI for each of the first four postoperative days (Table 2). PLR values on the day of discharge from hospital exhibited no association with the occurrence of AKI (Table 3). This shows that PLR values lost their significance beyond the first four days. Additionally, PLR exhibited the lowest AUC at ROC compared with NLR and delta-NLR, which restricts the use of PLR as a predictor of AKI (Table 6 and Figures 1, 2, and 3)

Lymphocyte counts were significantly lower in patients who developed AKI, they reached a nadir on postoperative day two (Table 2). Therefore, the predictive value of PLR 3 and 4 was reduced at univariate analysis (Table 5). Recent results show that PLR has a limited role as a predictor.

AKI is a complex entity, in which various injury mechanisms and injury sites may be present. The prediction of AKI in patients with normal preoperative creatinine values is important for treatment planning and to avoid further complications. Any novel biomarker should be able to show AKI in the first 72 hours after CABG, when creatinine levels are often nondiagnostic for AKI^8^. Various biomarkers have been proposed for predicting the occurrence and development of AKI in recent years. However, markers such as neutrophil gelatinase-associated lipocalin, cystatin C, IL-18, tissue inhibitor of matrix metalloproteinase-2, and insulin-like growth factor-binding protein 7 are expensive and not available in all hospitals^4,7^. NLR is, therefore, a useful tool in the prediction of AKI.

To the best of our knowledge, this is the first study demonstrating the use of dynamic changes in NLR in AKI prediction in patients undergoing CABG.

### Limitations

This is a retrospective study of a single center. The study was based on a relatively small sample size, especially the number of patients with AKI after postoperative day two diminished dramatically. The high NLR and PLR values obtained on days four and five may be associated with different postoperative factors either than the surgical stress itself. Due to the retrospective nature of the study, a causal relation could not be drawn. We employed the method used by Kertai et al.^13^ only to assess the relation between the continuous postoperative parameters with postoperative AKI. Instead of choosing a cutoff value for dichromatous analysis as originally described, we performed a ROC analysis to evaluate the predictivity and demonstrate the cutoff values of nadir and peak values as well as daily obtained parameters. We think this method provided a more detailed analysis.

## CONCLUSION

Augmented NLR and delta-NLR values were predictive for subsequent development of AKI on each of the first four postoperative days. NLR was the strongest and only independent predictor of in-hospital AKI. PLR lost its significant association with presence of AKI during hospital stay; therefore, we do not recommend its use beyond the first postoperative days. The detection of elevated NLR in the preoperative period as well as in postoperative days should serve as a warning for measures to be taken to treat kidney damage.

**Table:** 

Authors' roles & responsibilities	
HP	Substantial contributions to the conception or design of the work; and the acquisition, analysis or interpretation of data for the work; drafted the work or revised it critically for important intellectual content; agree to be accountable for all aspects of the work in ensuring that issues related to the accuracy or integrity of any part of the work are appropriately investigated and resolved; final approval of the version to be published
AAA	Substantial contributions to the conception or design of the work; and the acquisition, analysis or interpretation of data for the work; drafted the work or revised it critically for important intellectual content; agree to be accountable for all aspects of the work in ensuring that issues related to the accuracy or integrity of any part of the work are appropriately investigated and resolved; final approval of the version to be published
AÖ	Substantial contributions to the conception or design of the work; and the acquisition, analysis or interpretation of data for the work; drafted the work or revised it critically for important intellectual content; agree to be accountable for all aspects of the work in ensuring that issues related to the accuracy or integrity of any part of the work are appropriately investigated and resolved; final approval of the version to be published
